# Handbook of Massage

**Published:** 1893-07

**Authors:** 


HANDBOOK OF MASSAGE, by Emil Kleen, M. D., Ph. D. Authorized Translation from the Swedish, by Edward M. Hartwell, M. D., Ph. D. Philadelphia. P. Blakiston, Son & Co. Price, $2.75.

